# A Case of Idiopathic Multicentric Castleman’s Disease Diagnosed From Anemia and Renal Dysfunction on an Annual Check-Up

**DOI:** 10.7759/cureus.53472

**Published:** 2024-02-02

**Authors:** Sayato Fukui, Rikako Yokokura, Jura Oshida, Taisuke Kodama, Daiki Kobayashi

**Affiliations:** 1 General Internal Medicine, Tokyo Medical University Ibaraki Medical Center, Inashiki, JPN

**Keywords:** human herpesvirus 8, plasma cell, interleukin-6, hypercytokinaemia, anaemia, idiopathic multicentric castleman's disease

## Abstract

A 34-year-old man was referred to our hospital because of mild renal dysfunction and anemia. He had no specific preexisting medical conditions; his complaint was fatigue. Physical examination revealed several mobile, pinky head-sized (no tenderness) palpable lymph nodes on the bilateral neck. Blood biochemistry tests revealed anemia, renal dysfunction, increased inflammation, and a protein-albumin discrepancy. Immunological examination revealed polyclonal elevation of immunoglobulins (no shift in κ/λ ratio). A cervical lymph node biopsy was performed, and the pathological results showed numerous clusters of mature plasma cells (plasmacytic type), leading to the definitive diagnosis of idiopathic multicentric Castleman's disease (iMCD).

## Introduction

Castleman's disease (CD) is a rare lymphoproliferative disease. The CD was first reported as a disease of unknown cause by American pathologist Dr. Benjamin Castleman in 1956 and was named Castleman's disease [1]. CD is characterized by nonclonal lymph node hyperplasia [2]. It is classified as a hyaline, vascular, plasmacytic, or mixed cellularity type and may adopt a unicentric or multicentric presentation [2]. An association between the disease and human immunodeficiency virus (HIV) infection has been reported [2]. Human herpes virus 8 (HHV8) causes CD in immunosuppressed patients, including those infected with HIV (2). The cause of HHV8-negative multicentric CD is idiopathic and is called idiopathic multicentric Castleman's disease (iMCD) [3]. iMCD is a poorly understood lymphoproliferative disorder driven by hypercytokinemia. Patients have heterogeneous clinical features and characteristic lymph node histopathology. And multiple organ dysfunction occurs as it progresses [4]. In Japan, it is designated an intractable disease, and according to statistics from the Japan Intractable Diseases Information Center, the number of patients in Japan is approximately 1,500.

This case report describes a rare case in which the patient did not present to the hospital with multiorgan failure, as described above, but was diagnosed with renal dysfunction and anemia during an annual check-up.

## Case presentation

A 34-year-old man was referred to our hospital because of mild renal dysfunction and anemia during an annual check-up in 2023. The annual check-up in 2022 showed no renal function or anemia. However, the current blood test in 2023 showed creatinine of 1.37 mg/dL and hemoglobin of 8.5 g/dL.

He had no specific preexisting medical conditions; his chief complaint was fatigue. When he visited our hospital, his vital signs were normal, and a physical examination revealed several mobile, pinky-head-sized (no tenderness) lymph nodes on the bilateral neck. Other than this, no other obvious abnormal findings, including splenomegaly, were evident upon physical examination. Blood biochemistry tests revealed anemia, renal dysfunction, increased inflammation, and a protein-albumin discrepancy. Immunological examination revealed polyclonal elevation of immunoglobulins (no shift in κ/λ ratio) (Table 1).

**Table 1:** Blood and biochemistry test results HIV: human immunodeficiency virus

Blood test	Result	Normal range
White blood cells (/μL)	9,000	(4,000–8,000)
Neutrophils (%)	72.6	(40.0–75.0)
Lymphocytes (%)	15.9	(30.0–50.0)
Red blood cells (/μL)	3.31 × 10^6^	(4.35 × 10^6^–5.55 × 10^6^)
Hemoglobin (g/dL)	8.0	(12.0–16.0)
Hematocrit (%)	27.1	(37.0–47.0)
Mean corpuscular volume (μ)	81.9	(88.0–99.0)
Mean corpuscular hemoglobin (pg)	24.2	(29–35)
Mean corpuscular hemoglobin concentration (%)	29.5	(29–35)
Red cell distribution width (fL)	45.2	(35.2–50.5)
Reticulocyte (‰)	8.5	(5–20)
Platelets (/μL)	37.8 × 10^4^	(13.0 × 10^4^–35.0 × 10^4^)
Total protein (g/dL)	10.4	(6.7–8.3)
Albumin (g/dL)	2.6	(3.8–5.3)
Total bilirubin (mg/dL)	0.4	(0.2–1.0)
Aspartate aminotransferase (IU/L)	15	(12.0–32.0)
Alanine aminotransferase (IU/L)	15	(8.0–36.0)
Lactate dehydrogenase (IU/L)	127	(127.0–221.0)
Creatine kinase (IU/L)	47	(50.0–206.0)
Blood urea nitrogen (mg/dL)	14.6	(8.0–20.0)
Creatinine (mg/dL)	1.21	(0.65–1.07)
Sodium (mEq/L)	139	(134.0–147.0)
Potassium (mEq/L)	4.0	(3.2–4.8)
Chloride (mEq/L)	102	(98–108)
C-reactive protein (mg/dL)	7.90	(0.0–0.3)
Blood sedimentation rate (1 h value) (mm)	>140	
Prothrombin time (s)	15.0	(9.5–13.5)
Activated partial thromboplastin time (s)	34.8	(24.0–32.0)
Immunoglobulin G (mg/dL)	5196.1	(861–1747)
Immunoglobulin A (mg/dL)	689.3	(93–393)
Immunoglobulin M (mg/dL)	317.9	(33–183)
κ-chain (mg/L)	332	(3.3–19.4)
λ-chain (mg/L)	140	(5.7–26.3)
κ/λ ratio	2.37	(0.26–1.65)
Soluble interleukin receptor (U/mL)	2450	(157–474)
β2-microglobulin (mg/L)	3.89	(0–2)
HIV screening test	negative	negative

**Figure 1:**
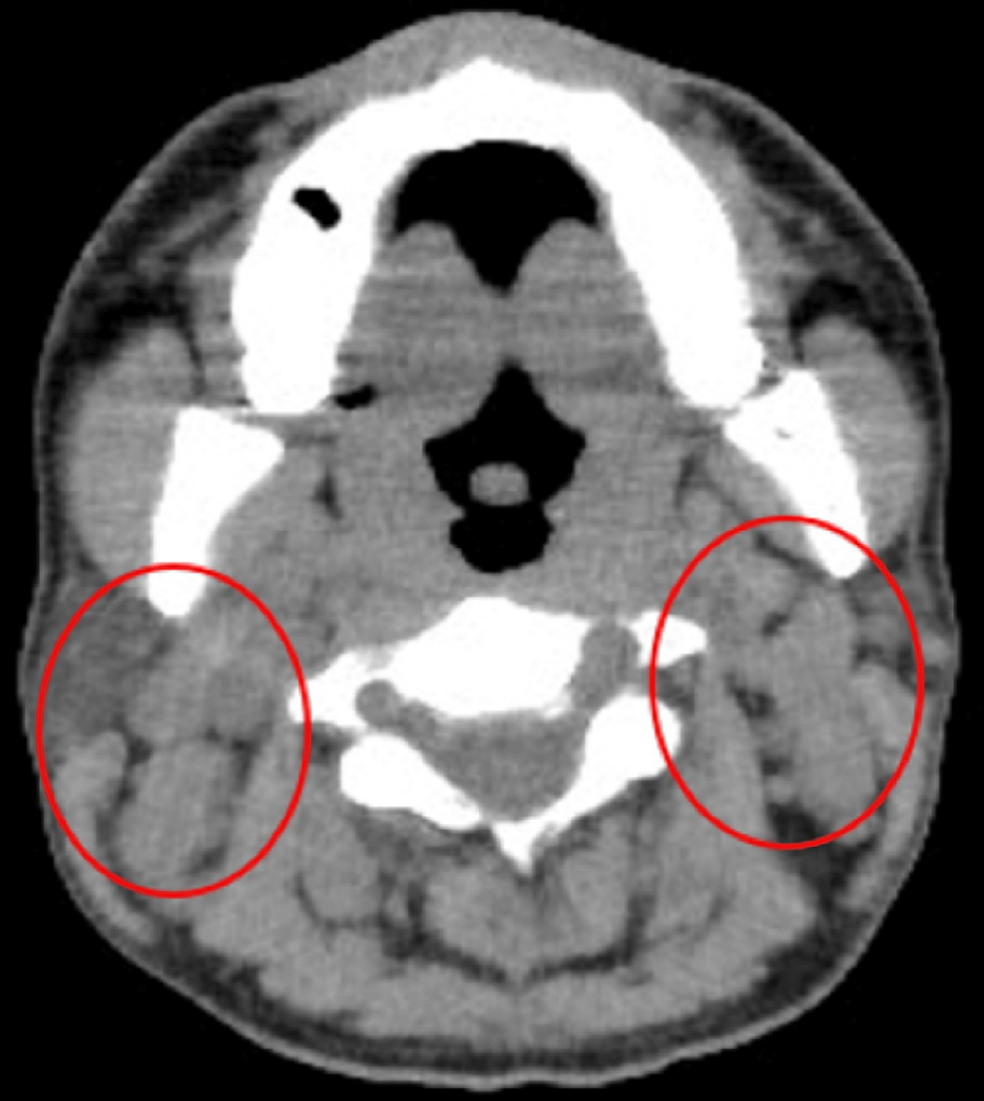
Computed tomography revealed multiple enlarged bilateral cervical lymph nodes (red circles)

A computed tomography (CT) scan showed multiple enlarged bilateral cervical lymph nodes (Figure 1).

**Figure 2:**
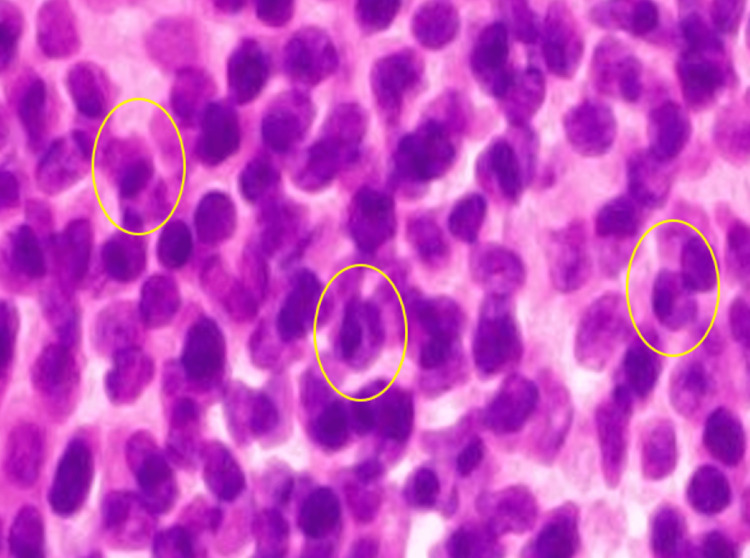
Cervical lymph node biopsy Plasma cells with nuclei predominantly located on one side were observed (inside the yellow circles) (Hematoxylin-Eosin stain; magnification ×400)

At this point, lymphoproliferative diseases, including Castleman's disease (CD) and malignant lymphoid species, were listed as differential diagnoses, and a cervical lymph node biopsy was performed. Pathological results showed numerous clusters of mature plasma cells (Figure 2).

**Figure 3:**
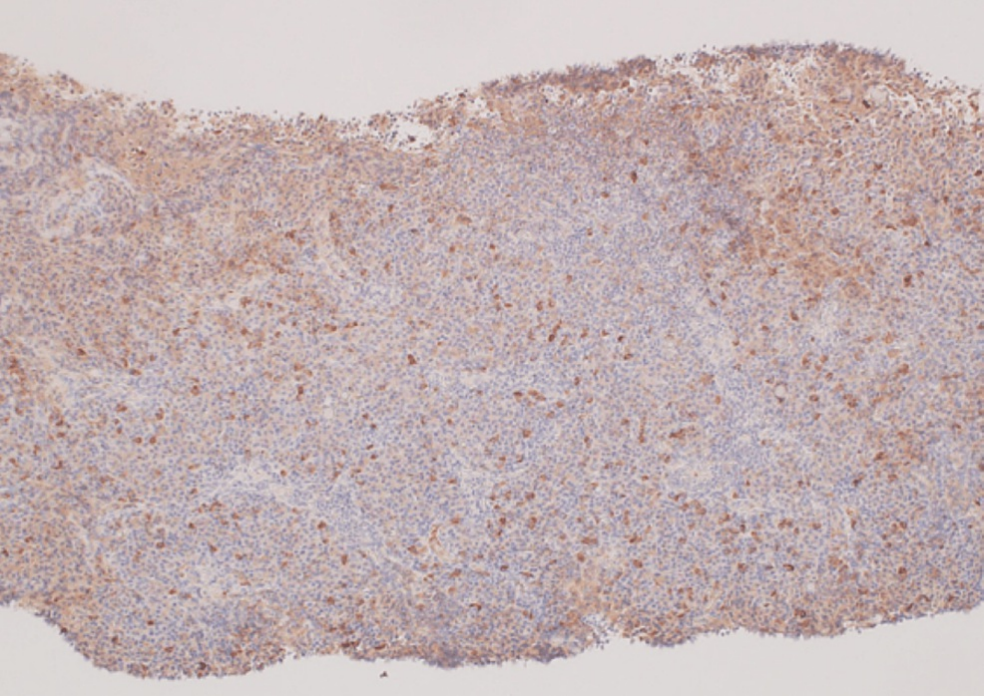
Immunoglobulin G immunostaining (overall stained) (×400)

**Figure 4:**
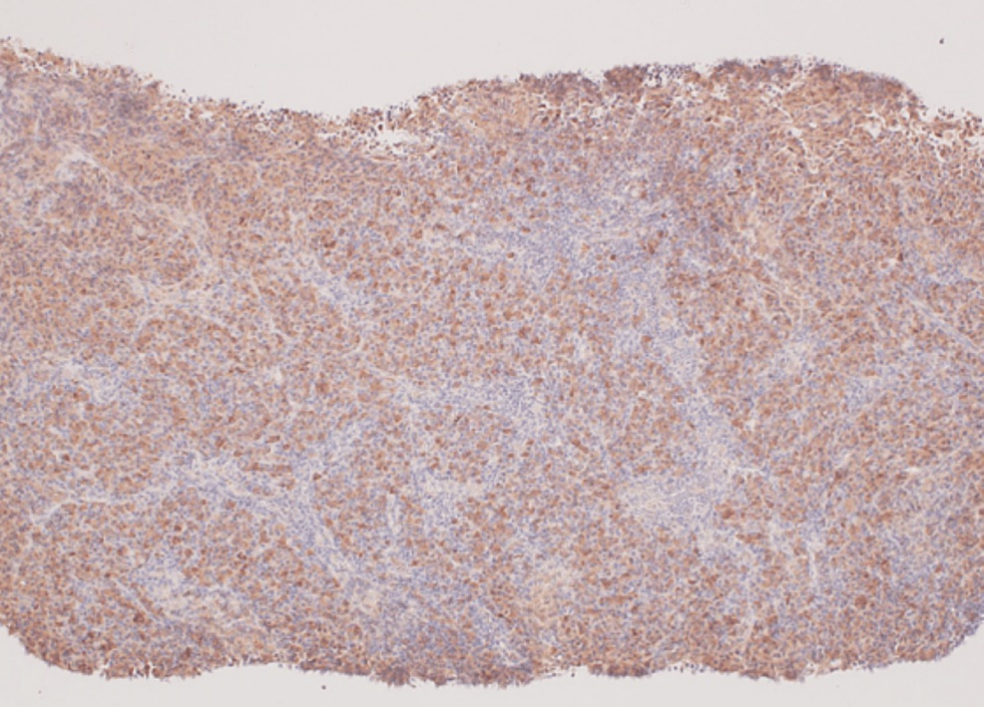
Light chain κ immunostaining (overall stained) (×400)

Immunostaining showed that all immunoglobulin staining was similar (no staining for specific immunoglobulins) (Figures 3, 4), ruling out plasmacytoma and suggesting multicentric Castleman's disease (plasmacytic type).

Subsequently, additional blood tests showed elevated interleukin-6 (IL-6); 23.4 pg/mL (normal value; <7.0 pg/mL). In addition, human herpesvirus type 8 (HHV-8) DNA quantification was below detection sensitivity (Table 2).

**Table 2:** Additional blood test results HHV: human herpes virus

Blood test	Result	Normal range
Interleukin-6 (IL-6) (pg/mL)	23.4	(<7.0)
HHV-8 DNA quantification (copy)	<2.0 × 10^1^	(<2.0 × 10^1^)

Furthermore, human immunodeficiency virus (HIV), autoimmune diseases, and malignant tumors were also excluded. These findings led to the definitive diagnosis of idiopathic multicentric Castleman's disease (iMCD).

## Discussion

Idiopathic multicentric Castleman's disease (iMCD) is a group of poorly understood lymphoproliferative disorders driven by hypercytokinaemia [4]. Patients have heterogeneous clinical features (characteristic lymph node histopathology and multiple organ dysfunction as it progresses) [4]. Although the progression of symptoms is thought to be gradual, there have been reports of cases diagnosed after severe disease, leading to multiple organ failure [5,6]. The present case was diagnosed based on minor abnormal findings during an annual check-up and was considered early. The only symptom was fatigue. The patient did not have any B symptoms, nor was he aware of the swollen lymph nodes in his neck. Castleman's disease (CD) would not have been included in the differential diagnosis without imaging if lymphadenopathy had not been detected on physical examination. This finding suggests the importance of a thorough physical examination in all cases.

The characteristic clinical findings of iMCD include multicentric lymphadenopathy, anemia, elevated C-reactive protein level, hypergammaglobulinemia, hypoalbuminemia, elevated IL-6, hepatomegaly or splenomegaly, fever, edema, ascites, anasarca, or a combination of these [4]. In our case, there was no fever or pleural effusion; therefore, we believe the patient did not notice any unusual symptoms. However, the blood test results were typical. Furthermore, if thrombocytopenia presents with marked pleural effusion, TAFRO (thrombocytopenia, anasarca (edema, pleural effusion, and ascites), fever, reticulin myelofibrosis (or renal insufficiency), and organomegaly (hepatosplenomegaly and lymphadenopathy)) syndrome should be considered a differential diagnosis. Because lymph node histology is similar between TAFRO syndrome with CD, TAFRO syndrome is described as related to CD [7].

Additionally, there are reports of biomarker studies in which C-X-C motif chemokine ligand (CXCL)-13 has been identified and validated as the protein most prominently upregulated in iMCD [8]. Furthermore, increases in inflammatory cytokines (serum IL-10 and IL-23) and chemokines such as CXCL-10 and vascular endothelial growth factor (VEGF)-A have been observed. And their relationship with pathogenesis has attracted attention [9]. The usefulness of these markers suggests that CD is caused by a cytokine storm. A few case reports are available in which adverse reactions to vaccines were thought to have elicited cytokine storms [10].

Treatment inevitably involves a therapy that suppresses cytokinesis. Cytokine-targeted therapy is the basis of treatment with corticosteroid monotherapy and IL-6-targeted therapy (tocilizumab or siltuximab) [4,11]. Our patient is currently undergoing treatment at this stage.

## Conclusions

We diagnosed the patient with idiopathic multicentric Castleman's disease (iMCD) at a relatively early stage. Diagnoses based on abnormalities during annual check-ups are rare. A detailed physical examination provided clues for the diagnosis.

On this day, and in the age of rapid developments in testing, including imaging tests, we were reminded again of the importance of detailed physical examination.
